# Characterisation of the normal human ganglion cell–inner plexiform layer using widefield optical coherence tomography

**DOI:** 10.1111/opo.13255

**Published:** 2023-11-22

**Authors:** Janelle Tong, David Alonso-Caneiro, Jason Kugelman, Jack Phu, Sieu K. Khuu, Michael Kalloniatis

**Affiliations:** 1 Centre for Eye Health, University of New South Wales, Sydney, New South Wales Australia; 2 School of Optometry and Vision Science, University of New South Wales, Sydney, New South Wales Australia; 3 School of Science, Technology and Engineering, University of Sunshine Coast, Sunshine Coast, Queensland Australia; 4 Contact Lens and Visual Optics Laboratory, Centre for Vision and Eye Research, School of Optometry and Vision Science, Queensland University of Technology, Kelvin Grove, Queensland Australia; 5 Faculty of Medicine, University of Sydney, Sydney, New South Wales Australia; 6 Concord Clinical School, Concord Repatriation General Hospital, Sydney, New South Wales Australia; 7 School of Medicine (Optometry), Deakin University, Victoria 3216, Waurn Ponds, Victoria Australia

**Keywords:** ageing, ganglion cell–inner plexiform layer, imaging, ocular coherence tomography

## Abstract

**Purpose:**

To describe variations in ganglion cell–inner plexiform layer (GCIPL) thickness in a healthy cohort from widefield optical coherence tomography (OCT) scans.

**Methods:**

Widefield OCT scans spanning 55° × 45° were acquired from 470 healthy eyes. The GCIPL was automatically segmented using deep learning methods. Thickness measurements were extracted after correction for warpage and retinal tilt. Multiple linear regression analysis was applied to discern trends between global GCIPL thickness and age, axial length and sex. To further characterise age-related change, hierarchical and two-step cluster algorithms were applied to identify locations sharing similar ageing properties, and rates of change were quantified using regression analyses with data pooled by cluster analysis outcomes.

**Results:**

Declines in widefield GCIPL thickness with age, increasing axial length and female sex were observed (parameter estimates −0.053, −0.436 and −0.464, *p*-values <0.001, <0.001 and 0.02, respectively). Cluster analyses revealed concentric, slightly nasally displaced, horseshoe patterns of age-related change in the GCIPL, with up to four statistically distinct clusters outside the macula. Linear regression analyses revealed significant ageing decline in GCIPL thickness across all clusters, with faster rates of change observed at central locations when expressed as absolute (slope = −0.19 centrally vs. −0.04 to −0.12 peripherally) and percentage rates of change (slope = −0.001 centrally vs. −0.0005 peripherally).

**Conclusions:**

Normative variations in GCIPL thickness from widefield OCT with age, axial length and sex were noted, highlighting factors worth considering in further developments. Widefield OCT has promising potential to facilitate quantitative detection of abnormal GCIPL outside standard fields of view.

**Supplementary Information:**

The online version of this article (doi:10.1111/opo.13255) contains supplementary material, which is available to authorized users.

## Key points

- This study described the generation of ganglion cell–inner plexiform layer thickness measurements from a widefield optical coherence protocol, spanning 55° × 45° across the central retina.
- Characterisation of age, axial length and sex-related changes in the widefield ganglion cell–inner plexiform layer from healthy eyes can facilitate the detection of abnormal, possibly pathological changes.
- Capturing optical coherence data across retinal locations sampled using common visual field protocols also enabled more direct assessment of the structure–function relationship.

## INTRODUCTION

With the advent of optical coherence tomography (OCT) imaging, the ability to visualise individual retinal layers in vivo has revolutionised ophthalmic clinical care.^1^ In particular, the automated measurement of retinal thickness parameters and concurrent quantitative comparisons to normative data enables the quick and reliable identification of various ocular pathologies.^2–4^ However, most commercially available OCT devices utilise scan protocols that are restricted to the macular and peripapillary regions, limiting the current applications of quantitative OCT data to pathologies affecting relatively central retinal locations.

Scan protocols covering larger retinal areas have recently been developed to enable rapid visualisation of non-central locations without requiring additional scan acquisition. The term widefield OCT is used to describe several scan protocols of this nature, with this label most commonly referring to scans spanning 12 mm × 9 mm (approximately 41.67° × 31.25°) available in devices such as the Topcon DRI OCT Triton (global.topcon.com). Widefield OCT studies have reported equivalent or superior detection of glaucomatous damage compared to conventional scan protocols,^5–8^ and the greater number of captured OCT locations enabling more direct comparisons to co-localised visual field data is a purported key advantage of this protocol.^9^ However, this widefield OCT protocol does not capture up to 50% of retinal locations matching the 24-2 visual field test grid, which is commonly used in clinical investigations of visual function.^10^

Recently, OCT protocols covering the central 55° × 45° of the retina in a single scan have been demonstrated to return ganglion cell–inner plexiform layer (GCIPL) thickness measurements equivalent to those of conventional OCT protocols.^11,12^ This suggests that reliable acquisition of OCT data matching retinal locations sampled with standard visual field paradigms is certainly possible. However, due to its relative recency, a holistic understanding of how quantitative measurements derived from widefield OCT vary within healthy cohorts is lacking. In studies using central OCT protocols, variations in retinal thickness secondary to age, refractive error, axial length (AL), sex and ethnicity have been described,^13–21^ suggesting that compensation for these factors may be required before accurate comparisons to normative database measurements can be performed. An exploration of whether demographic variables similarly affect retinal measurements acquired using widefield OCT protocols would be valuable to identify potential confounders that could affect the accurate identification of pathology, and therefore ensure that subsequent normative databases are sufficiently representative.

In this study, GCIPL thickness measurements were extracted from widefield OCT scans, spanning 55° × 45°, acquired from a healthy cohort. The GCIPL was chosen as the retinal complex of interest due to its histological correlation with the retinal ganglion cells and their dendritic processes,^22,23^ which have been studied extensively to characterise the relationship between retinal structure and visual field data.^24–27^ While the RNFL has been applied in similar contexts,^9,28,29^ it is difficult to determine with OCT imaging alone whether the RNFL at a particular location reflects axons from co-localised ganglion cells, or a continuation of axons from relatively distal ganglion cells. This uncertainty, in conjunction with the propensity for inter-individual variations in RNFL trajectory,^30,31^ renders the GCIPL alone a potentially less error-prone structural correlate for use in analyses where co-localisation with other data is pertinent. The potential influences of various demographic factors on GCIPL thickness measurements were analysed, with age in particular hypothesised to impact GCIPL thicknesses significantly as reported by previous studies of the macula.^13,14,17,32^ Detailed investigations of factors influencing GCIPL thickness measurements acquired using widefield OCT could support its clinical translation, with potential applications including the detection of extra-macular ocular pathologies and comparisons to visual field results across relatively peripheral locations.

## METHODS

### Participant recruitment

Participants were prospectively recruited from patients attending the Centre for Eye Health (Sydney, Australia), and staff and students from the Centre for Eye Health and the School of Optometry and Vision Science, University of New South Wales (Sydney, Australia). As per clinical examination protocols at the Centre for Eye Health,^33–35^ participants underwent slit-lamp biomicroscopy, funduscopic examination, applanation or rebound tonometry (iCare tonometer; Icare Finland Oy, icare-world.com), and optic disc and macular OCT (Cirrus HD-OCT; Carl Zeiss Meditec Inc., zeiss.com/corporate/en/home.html). Additional assessments performed on indication, such in specific examinations for suspected glaucoma, included keratometry and AL measurement (IOL Master; Carl Zeiss Meditec, zeiss.com/corporate/en/home.html), central corneal thickness measurement (Pachmate; DGH Technology, dghtechnology.com), applanation tonometry, gonioscopic examination of the anterior chamber angle and visual field assessment using the 24-2 Swedish Interactive Threshold Algorithm Faster paradigm (Humphrey Field Analyzer; Carl Zeiss Meditec, zeiss.com/corporate/en/home.html). Participants' demographic information was extracted from either patient intake forms administered prior to clinical appointments or self-reported by staff and students.

As reported by previous studies,^13,14,32^ inclusion criteria were ≥20 years of age, spherical equivalent refractive error between +6.00 and −6.00 dioptres, no more than −3.00 dioptres of astigmatism, intraocular pressures <22 mmHg in both eyes, no macular pathology in at least one eye and no optic nerve pathology in either eye based on the results of funduscopic examination and OCT imaging. Where one eye met these inclusion criteria, this eye was selected for subsequent analyses, while if both eyes met the inclusion criteria then one eye was chosen at random to prevent potential for correlated data between eyes of the same participants. For patients attending the Centre for Eye Health, the absence of ocular pathology was confirmed by two optometrists in accordance with clinical protocols.^33,36^ While the Centre for Eye Health is a referral-based clinic for patients suspected of ocular pathology, in a recent audit 22.6% of patients were subsequently found to have no ocular pathology.^33^ All participants provided written informed consent to participate in this study, as per the ethics protocols approved by the University of New South Wales Human Research Ethics Advisory Panel, and the study adhered to the tenets of the Declaration of Helsinki throughout its duration.

### Widefield OCT acquisition

Widefield OCT scans were acquired using the Widefield Imaging Module on Spectralis OCT (Heidelberg Engineering, heidelbergengineering.com/int/). The volume scan protocol applied in this study acquires 109 B-scans spaced 120 μm apart at a scan tilt of 0°, spanning a total area of 55° horizontally and 45° vertically.^11^ Each B-scan was averaged nine times, and participants were instructed to maintain central fixation during scan acquisition. Scans that did not meet a minimal signal strength requirement of 15 dB were excluded from further analyses. Additionally, all B-scans were manually examined for focal reductions in signal strength and motion artefacts, with individual B-scans excluded as required.

Where available, mean keratometry readings were input into the instrument review software (HEYEX; Heidelberg Engineering, heidelbergengineering.com/int/) to adjust for potential transverse magnification effects; in cases where keratometry readings were unavailable, this was set to the instrument standard of 7.7 mm. For each participant, individual B-scans and the corresponding scanning laser ophthalmology (SLO) image were exported as image files, with further scan information including lateral resolution in millimetres per pixel extracted from the corresponding Extensible Markup Language (XML) file.

### Widefield OCT segmentation and processing for GCIPL measurements

**FIGURE 1:**
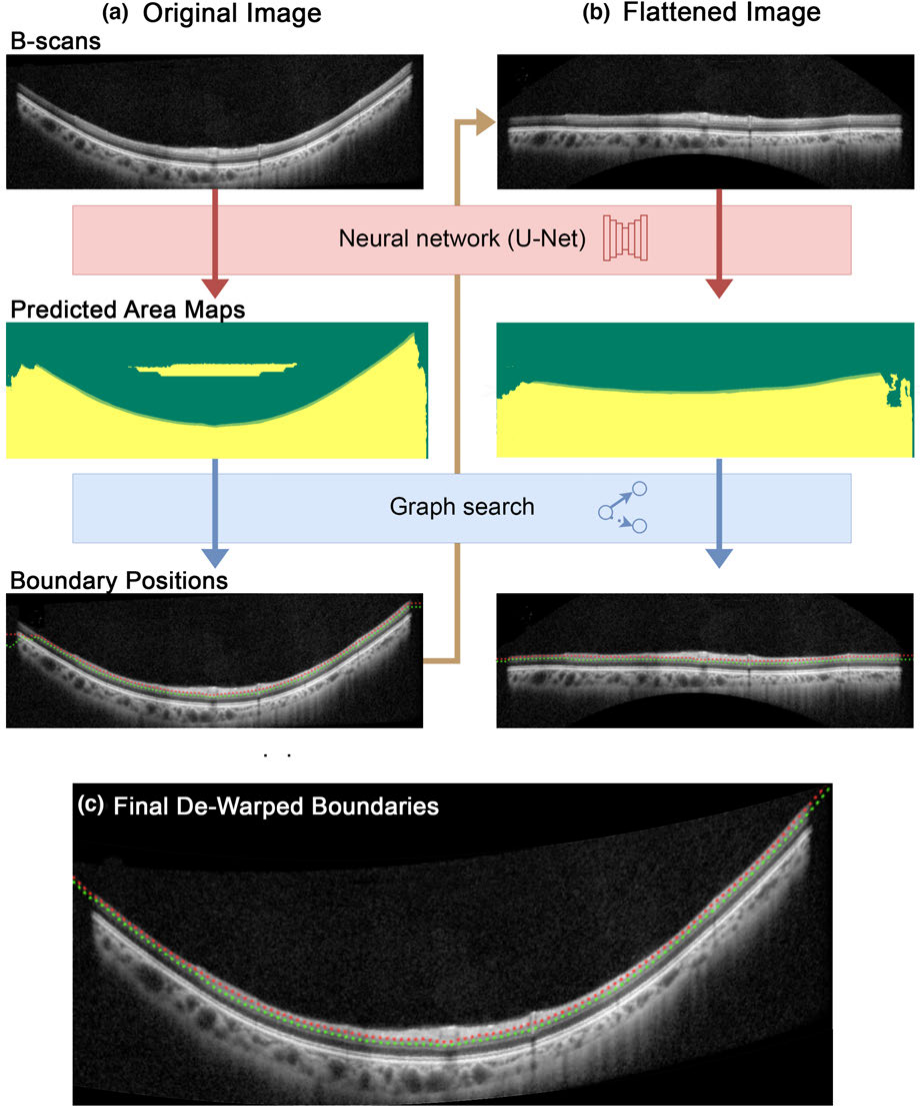
Overview of the segmentation pipeline using the deep neural network and graph search for an example optical coherence tomography (OCT) B-scan. (a) The original image was passed through the neural network to produce predicted area maps, where the dark green and yellow areas correspond to different regions of interest predicted per pixel. Graph search was used to infer the positions of the retinal nerve fibre layer–ganglion cell layer (RNFL–GCL) (red) and inner plexiform layer–inner nuclear layer (IPL–INL) (green) boundaries. (b) To verify the boundaries predicted in A, the original OCT image was also flattened and the process in A repeated. (c) The final RNFL–GCL and IPL–INL boundaries after reversing the flattening process and de-warping the OCT scan to correct post-acquisition flattening.

A segmentation pipeline using a deep learning approach was applied to segment the two retinal layer boundaries delineating the GCIPL, the retinal nerve fibre layer–ganglion cell layer (RNFL–GCL) and inner plexiform layer–inner nuclear layer (IPL–INL) boundaries (Figure 1). In brief, this consisted of cropped OCT images, used to improve generalisability to unseen OCT data, input into a typical encoder–decoder neural network (U-Net).^37,38^ The output of this process was the per-pixel classification of OCT B-scan images into one of three classes, representing the regions separated by the RNFL–GCL and IPL–INL boundaries, which was subsequently used to infer boundary locations via graph search. This process was first applied with the raw widefield OCT image to produce initial predicted layer boundary positions, which were subsequently refined by repeating the process using a flattened OCT image, to normalise the appearance of the retinal tissue structure in the image provided to the network.^37^ This was performed by fitting a second-order polynomial to the IPL–INL boundary, and A-scans were translated using this polynomial to flatten the OCT image prior to re-input to the network and a subsequent graph search. The new boundary positions were then transformed to their original coordinates by undoing the flattening to give the segmented RNFL–GCL and IPL–INL boundaries. Further technical details of the segmentation process are described in Supporting Information S1.

From here, gross errors in segmentation were manually corrected, while uncorrectable segmentation errors due to blood vessel shadowing, encroachment of the optic disc or peripapillary atrophy and focal loss of signal strength were manually delineated and excluded. Additionally, the scanning laser ophthalmoscopy (SLO) images were binarised to demarcate locations of the retinal vasculature and complement the manual delineation process.

**FIGURE 2:**
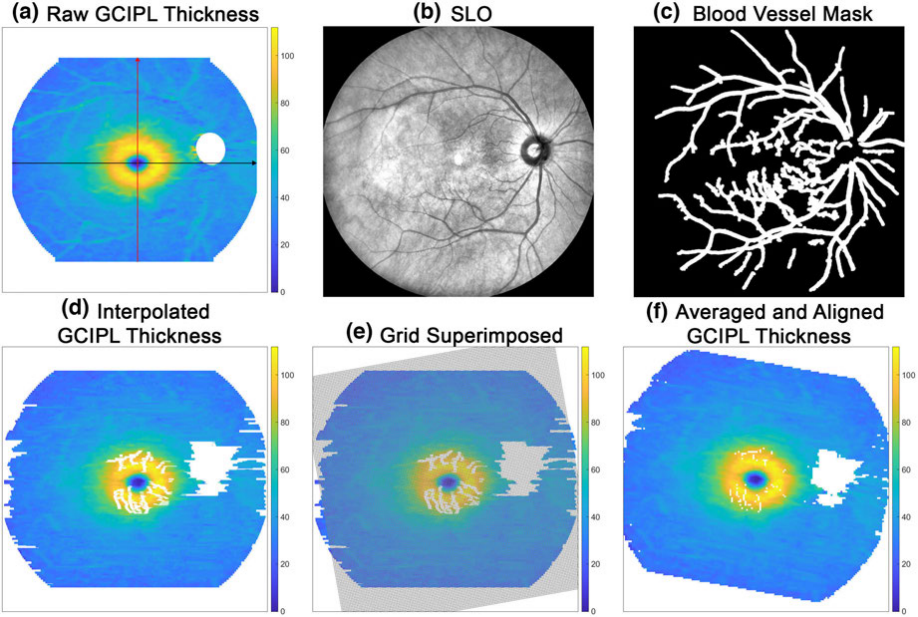
Overview of ganglion cell–inner plexiform layer (GCIPL) thickness map generation processes. Coloured bars throughout indicate GCIPL thickness in microns. (a) GCIPL thickness measurements across the total scan area after correction for retinal tilt. Curvature of the foveal B-scan (black arrow) was used to calculate curvature along the vertical meridian (red arrow), enabling translation of B-scans to reconstruct the posterior globe shape. (b) The scanning laser ophthalmoscopy (SLO) image used to generate the (c) binary blood vessel mask to complement the manual identification of uncorrectable segmentation errors. (d) GCIPL thickness measurements were linearly interpolated within regions of uncorrectable segmentation error. Exceptions were locations adjacent to the optic disc, within the macular region and at the scan edges, which remained excluded. (e) A 160 × 160 grid aligned to the fovea to optic disc tilt, with grid squares measuring 100 × 100 μm, over which (f) GCIPL measurements were averaged for subsequent analysis.

The segmented RNFL–GCL and IPL–INL boundaries were further processed using software coded in Matlab Version 2021b (Mathworks, au.mathworks.com, Figure 2). First, bicubic interpolation was used to resize the dimensions of the segmented boundary maps to those of the SLO images.^39,40^ As OCT B-scans were acquired along the horizontal meridian, to ensure each B-scan was captured fully within the scanning area, there is often a loss of curvature information along the vertical meridian. B-scans were therefore translated along the z-axis based on estimates of vertical curvature, calculated from the refractive error (rx) of the scanned eye and the long-axis parameter from an elliptical function fitted to the foveal B-scan, which was manually selected from B-scan images^41^:

1 $$ \mathrm{axi}{\mathrm{s}}_{\mathrm{vert}}=\left(-0.004072\times \mathrm{rx}+0.9921\right)\times {\mathrm{axis}}_{\mathrm{horiz}} $$

Eyes where an elliptical function could not be fitted to the foveal B-scan were excluded from subsequent analyses, as it was inferred that the presence of an atypical macular profile, for example a macular posterior staphyloma, would not be suitable for inclusion in analyses of normal eyes.

Boundaries were then de-warped to compensate for OCT-related flattening of B-scan images affecting interpretation of retinal curvature,^42–44^ by adjusting boundary location along the *z*-axis to remain equidistant from the nodal point of the eye, the position of which was calculated from a combination of AL and refractive error data.^45^ For eyes that did not have AL data directly available, due to the moderate correlation found using Pearson correlation analyses (*R* = −0.65),^46^ this was calculated from refractive error per linear regression analyses (described in further detail in Supporting Information S2):

2 $$ \mathrm{axial}\ \mathrm{length}=-0.412\times rx+23.757 $$

While GCIPL thickness is typically derived from the axial distance between the RNFL–GCL and IPL–INL boundaries, axial measurements typically over-estimate retinal thickness with increasing retinal tilt,^11,44,47–49^ which becomes more pertinent with widefield OCT given greater retinal tilt with increasing eccentricity. As such, axial GCIPL thicknesses and retinal tilt relative to the foveal centre were calculated across the total scan area and adjusted to derive corrected GCIPL thicknesses perpendicular to the IPL–INL boundary, per the following equation (the derivation of which is described in Supporting Information S3):

3 $$ {\displaystyle \begin{array}{c}\mathrm{GCIP}{\mathrm{L}}_{\mathrm{corrected}}=\mathrm{GCIP}{\mathrm{L}}_{\mathrm{uncorrected}}-{14.03}^{\ast } til{t}^2\hfill \\ {}\kern6.5em +1.611\times tilt-0.07437\hfill \end{array}} $$

with GCIPL thicknesses measured in microns and retinal tilt in radians. Then, corrected GCIPL thickness measurements were used to linearly interpolate GCIPL thickness along the horizontal meridian at locations that were previously excluded due to uncorrectable segmentation, delineated manually and with the blood vessel mask. Due to greater potential for error in interpolation at locations adjacent to the optic disc, within the macular region where the largest variation in GCIPL thickness was observed, and at the scan edges at these locations any areas flagged for exclusion in prior processing stages remained excluded. Where entire B-scans had been excluded, interpolation across this missing area was not performed.

While HEYEX calculates transverse magnification as a function of corneal curvature and SLO image focus, related to refractive error, axial length has also been identified as contributing to transverse magnification,^50^ and without compensation may lead to non-corresponding retinal areas being compared between participants. As such, the lateral resolution values extracted from XML files were corrected for individual participants' axial length using the following equation:

4 $$ {\displaystyle \begin{array}{c}\mathrm{Corrected}\ \mathrm{lateral}\ \mathrm{resolution}=\hfill \\ {}\kern1em \mathrm{Original}\ \mathrm{lateral}\ \mathrm{resolution}\times \frac{\mathrm{Axial}\ \mathrm{length}}{24.385}\hfill \end{array}} $$

To equalise scaling factors between participants, ensuring corresponding locations were compared between participants, and facilitate further analyses while retaining a sufficient sampling resolution, GCIPL measurements were averaged across grid squares measuring 100 × 100 μm, similar to previously described methods,^51,52^ over a total grid area of 160 × 160 grid squares. While this exceeded the total scan area for most participants, this was performed to ensure all data were captured in averaged grid square measurements. Grid tilt was also aligned using individual participants' fovea to optic disc tilt,^7,39,40,53^ calculated from the foveal and optic disc centres manually selected from B-scan and SLO images, respectively. The grid was then rotated such that the grid tilt was 0°, to ensure that corresponding locations were grouped together in subsequent analyses.

### Statistical analysis

Statistical analyses were performed using SPSS Statistics Version 27.0 (ibm.com) and GraphPad Prism Version 8.4.3 (graphpad.com). To determine the suitability of using axial length measurements predicted from refractive error, the repeatability coefficient (*C*_*R*_) was calculated between corrected GCIPL measurements using measured axial lengths (GCIPL_measured_) versus those calculated from refractive error (GCIPL_calculated_) for a subset of 60 randomly selected eyes (Table S1), using the following equation^54^:

5 $$ {C}_R=1.96\times \sqrt{\frac{\sum \left|\mathrm{GCIP}{\mathrm{L}}_{\mathrm{measured}}-\mathrm{GCIP}{\mathrm{L}}_{\mathrm{calculated}}\right|}{n}} $$

In the above equation, GCIPL measurements were averaged across grid squares, with *n* representing the total number of grid squares of GCIPL thickness included in this calculation.

Multiple linear regression analyses were performed to identify demographic features impacting global GCIPL thickness measurements captured using widefield OCT, and that could therefore impact normative database comparisons. GCIPL thickness measurements averaged across the 160 × 160 grid for each participant were set as the dependent variable, while age, axial length and self-reported sex (binarised to male as 0 and female as 1) were set as main effects. While GCIPL measurements were corrected for axial length-related transverse magnification effects, the multiple linear regression analyses sought to identify whether additional effects of axial length on GCIPL thickness measurements were present. Refractive error was not included in this analysis due to its moderate correlation with axial length (Supporting Information S2), and violation of independence of main effects could confound the multiple linear regression model. To mitigate potential over-fitting,^55,56^ a process akin to backwards stepwise elimination was used, where the least significant main effect was removed and the remaining effects were reanalysed; should the difference between the original and reanalysed coefficients exceed 10%, the removed effect was deemed to be sufficiently impactful and therefore retained in the model.^57^

Given previous reports of variable, non-linear rates of age-related change in GCL thickness from the central to peripheral macula,^13,14,32^ further investigations of whether similar trends occur in GCIPL measurements captured over the central 55° × 45° are warranted. Cluster analysis enables location-agnostic identification of subsets of data demonstrating similar statistical characteristics and has been previously applied to describe patterns of age-related change in visual field, corneal, OCT and OCT angiography parameters,^32,52,58–63^ and visual field progression in glaucoma.^64–67^ In this study, we used similar approaches to identify locations demonstrating similar ageing properties of GCIPL captured using widefield OCT, and those that were suitable to pool together in subsequent analyses to redistribute measurement variability. Prior to cluster analysis, participants were grouped into age brackets (20 to <30 years, 30 to <40 years up to 70+ years). Grid-wise GCIPL thickness measurements were corrected for demographic factors other than age per the multiple linear regression model, and corresponding grid square locations were averaged across participants in each age bracket.

Two cluster algorithm approaches were applied in the present study. Hierarchical cluster analysis is an agglomerative procedure where each data point begins as a discrete cluster and is consecutively merged together in the order of the most to the least similar based on squared Euclidean distance and within-groups linkage until an endpoint of one cluster containing all data points is achieved.^13,59,60,62^ Two-step cluster analysis first coarsely groups data into clusters based on a distance measure, such as Euclidean distance, and these are subsequently refined using a probability-based approach to group these into the final desired number of clusters.^68,69^ An advantage of both approaches over other algorithms is that they do not require *a priori* assumptions on the number of most suitable clusters, which can instead be determined using statistical criteria such as minimum separability between clusters. However, due to the volume of data owing to the high sampling density applied in this study, there is the potential for an excessive number of clusters, or over-assignment using conventional separability criteria.^51,70,71^ As such, the silhouette coefficient was derived from calculations of the distance between data points within a cluster and to data points in the nearest neighbouring cluster, and a minimum silhouette coefficient of 0.5 was used to determine the maximum number of suitable clusters,^72,73^ which was subsequently applied as a criterion in the hierarchical cluster analysis. Distance measures applied between clusters were Euclidean distance for two-step cluster analysis and squared Euclidean distance for hierarchical cluster analysis, while within-groups linkage was applied in hierarchical cluster analysis to determine similarity within clusters. Across both methods, per previous applications of cluster analysis,^13,59,60,62^ a minimum of 1, indicating at least 1 standard deviation separating cluster means, was used to determine suitable cluster separability, from d′ measurements calculated across cluster pairs based on the mean (*x*) and standard deviation (*σ*) for each cluster:

6 $$ {d}^{\prime }=\frac{\mid {x}_1-{x}_2\mid }{\sqrt{0.5\times \left({\sigma}_1^2+{\sigma}_2^2\right)}} $$

Clusters derived using two-step and hierarchical cluster analysis were subsequently used to pool data prior to further regression analysis, to characterise change in GCIPL thickness across the widefield OCT measurement area as a function of age. To account for within-eye correlations due to the inclusion of multiple clustered GCIPL measurements per eye, a linear mixed effects model was applied, with GCIPL thickness averaged across each cluster set as the dependent variable, age, cluster identity and their interaction set as the fixed effects, and subject eye set as a random effect. Moreover, as absolute rates of change may be proportional to peak GCIPL thickness measurements,^13,32^ linear mixed effect models were also applied with GCIPL thickness data transformed to a logarithmic scale (log(GCIPL thickness)) to enable visualisation of percentage change in GCIPL thickness as a function of age. From these models, the derived estimated marginal means, which keeps the random effect constant across all participants and therefore accounts for the potential confounding related to data from the same eyes contributing to multiple clusters, were applied to subsequent regression analyses.

High inter-individual variability in retinal thickness measurements, as previously observed within macular OCT-based studies,^13,14^ could result in excessive noise masking notable trends when using pointwise data. While noise-related concerns can be mitigated by data pooling, to ensure that the chosen grouping by age brackets did not bias the regression model outcomes, sliding window analysis was performed on GCIPL thickness data to aid regression model choice.^60,62,74^ Maintaining decade-sized windows, 1 year was sequentially added to the start of each age bracket (that is 21 to <31 years, 31 to <41 years up to 71+ years, then 22 to <32 years, 32 to <42 years up to 72+ years and so forth). Gridwise GCIPL thickness measurements falling within each cluster were averaged across participants falling within each window and plotted against the mean age for the corresponding window. Quadratic and linear regression models to the sliding window data were applied to each cluster, and extra sum-of-squares *F* test and adjusted coefficient of determination (*R*^2^) comparisons were performed to directly contrast models.

From outcomes of sliding window analysis, the simplest model producing a meaningful improvement in model fit was chosen for regression analysis on data pooled using the original age brackets and each cluster. For both GCIPL thickness and log(GCIPL thickness), to determine whether a single set of regression coefficients could adequately describe age-related changes across all clusters, likelihood ratio test statistics and *F* statistics were calculated between the original models with unconstrained coefficients and a simpler model constraining a single set of regression coefficients across all clusters. For the former, this required additional linear mixed effects models to be computed with the interaction term between age and cluster identity removed. However, as this approach does not provide information on whether specific groups of clusters may share regression coefficients, post-hoc comparisons of regression coefficients between cluster pairs were performed using analyses of variance with Tukey's multiple comparisons tests with corrections for multiple comparisons, the results of which were used to inform groups of clusters to calculate F statistics as above. Throughout this study, the threshold for statistical significance was set at *p* < 0.05.

## RESULTS

### Participant demographics

Originally, 476 eyes of 476 healthy participants were eligible for inclusion in this study. After widefield OCT scan processing, six eyes were excluded due to a presumed atypical macular profile, resulting in 470 eyes comprising the final study cohort. Demographic features of the entire cohort, as well as divided into age brackets utilised for cluster and regression analyses, are provided in Table 1.

**TABLE 1:** Demographic features of the study cohort, including division by age brackets used in cluster and regression analyses.

	*n*	Age (y ± SD)	Sex (M:F)	SE (D ± SD)	AL (mm ± SD)	Eye (OD:OS)	Tilt (° ± SD)	IOP (mmHg ± SD)	Ethnicity (White:East Asian:Other)
All	470	51.29 ± 16.92	208:262	−0.42 ± 1.88	23.97 ± 1.12	247:223	7.14 ± 3.87	14.64 ± 3.00	240:143:87
By age bracket (years)									
20 to <30	70	24.93 ± 2.83	25:45	−1.05 ± 1.37	24.42 ± 0.98	39:31	5.84 ± 3.53	14.38 ± 4.69	20:37:13
30 to <40	73	35.07 ± 2.85	34:39	−1.44 ± 1.94	24.26 ± 1.53	31:42	7.07 ± 3.26	14.89 ± 3.09	29:29:15
40 to <50	70	45.32 ± 2.49	28:42	−0.83 ± 1.86	23.81 ± 1.19	32:38	7.27 ± 4.01	14.70 ± 2.97	40:14:16
50 to <60	80	55.34 ± 3.07	32:48	−0.42 ± 1.72	23.97 ± 1.28	42:38	6.63 ± 4.13	14.41 ± 2.65	36:28:16
60 to <70	102	65.05 ± 2.95	44:58	0.25 ± 1.89	23.94 ± 1.15	61:41	7.53 ± 3.77	14.84 ± 2.96	58:25:19
70+	75	74.21 ± 3.39	45:30	0.62 ± 1.60	23.62 ± 0.92	42:33	8.28 ± 4.12	14.10 ± 3.22	57:10:8

*Note*: Axial lengths reported include those measured (*n* = 345) and calculated from correlation analyses (*n* = 125, Supporting Information S1). For brevity, participants self-identifying as ethnicities other than White or East Asian are pooled together as Other; the complete distribution of ethnicities is provided in Table S2.

Abbreviations: °, degrees; AL, axial length; D, dioptre; F, female; IOP, intraocular pressure; M, male; *n*, number of participants; OD, right eye; OS, left eye; SD, standard deviation; SE, spherical equivalent refractive error; y, years of age.

Of note, due to the demographic characteristics of patients attending the Centre for Eye Health, the majority of participants self-identified as White or East Asian ethnicity, with far fewer participants self-identifying as Aboriginal or Pacific Islander, African, Central or South American, South Asian or Middle Eastern (Table S2).

### Use of calculated axial length

From the subset of 60 eyes where comparisons between GCIPL measurements corrected using measured axial lengths and those calculated from correlation analyses using refractive error were performed, *C*_*R*_ between these GCIPL measurements was 2.41 μm. The axial resolution, the micron measurement associated with 1 pixel's worth of error in segmentation, of the Spectralis OCT was 3.87 μm.^11^ As this *C*_*R*_ value fell well within this axial resolution, axial lengths predicted from refractive error were suitable for the derivation of widefield GCIPL thicknesses as per the utilised study protocol.

### Demographic variables influencing widefield GCIPL thickness

Multiple linear regression analyses were applied to investigate the effects of age, axial length and sex on averaged global GCIPL thickness across the widefield OCT measurement area, which revealed significant effects across all investigated demographic variables (Table 2). With the removal of sex as the least significant variable from the regression model, the difference in age and axial length coefficients was 5.66% and 42.57%, respectively. Since the difference in the axial length coefficient exceeded 10%, this indicated that all demographic variables impacted global GCIPL thickness sufficiently. Overall, these coefficients indicated a decline in global GCIPL thickness with age, increasing axial length and female sex.

**TABLE 2:** Coefficients derived from multiple linear regression analyses, with all demographic variables included and sex excluded.

Variable	All variables included		With sex removed		
	Parameter estimate ± SE	*p* Value	Parameter estimate ± SE	*p* Value	Difference (%)
Age	−0.053 ± 0.008	<0.001	−0.050 ± 0.008	<0.001	5.66
Axial length	−0.436 ± 0.124	<0.001	−0.371 ± 0.121	0.002	42.57
Sex	−0.646 ± 0.273	0.02	–	–	–

Abbreviation: SE, standard error.

Due to the paucity of participants self-identifying as ethnicities other than White or East Asian, the study was underpowered to investigate the potential for ethnicity to influence GCIPL thickness. However, to examine the effect of White or East Asian ethnicity, multiple linear regression analyses were repeated with these participants only, which did not reveal a significant effect (β = 0.043 ± 0.294, *p* = 0.88, Table S3).

### Cluster analysis to determine spatial ageing changes in the GCIPL

**FIGURE 3:**
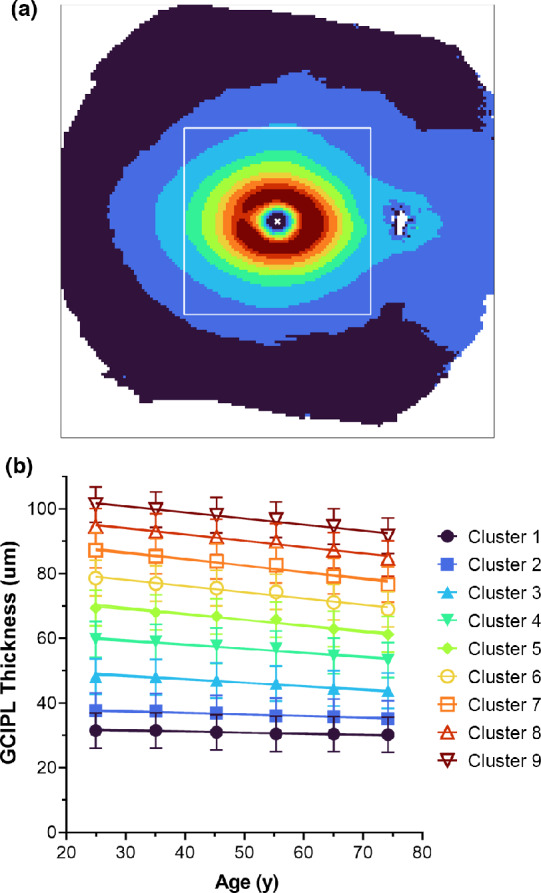
(a) Spatial patterns of age-related change in ganglion cell-inner plexiform layer (GCIPL) thickness across the central 55° × 45° of the retina, generated using the hierarchical cluster algorithm. Different colours denote statistically separable clusters of increasing GCIPL thickness. The white box demarcates the posterior pole volume scan area using the Spectralis optical coherence tomography (OCT), as described in previous cluster analysis methods,^13,14,32^ to enable visualisation of the additional retinal area captured using widefield OCT. (b) Linear regression model describing GCIPL thickness pooled by cluster as a function of age. The differently coloured regression lines match the coloured locations in (a). Equations and fit parameters can be found in Table 4. Given the overall similarities between hierarchical and two-step cluster algorithms, results for the two-step cluster algorithm can be found in Figure S1.

After correction of grid square GCIPL thickness measurements for sex and axial length, cluster analyses were applied to data pooled by decade bracket, to aid visualisation of spatial patterns for age-related changes in GCIPL measurements across the retinal locations scanned with widefield OCT. A maximum of nine statistically separable clusters were found to meet both silhouette and d′ criteria. Using both hierarchical and two-step cluster algorithms, concentric, horseshoe patterns of age-related change in the GCIPL were observed, with a reasonably symmetrical distribution of GCIPL measurements within the macula but a slight nasal displacement outside the central 25° of the retina; thus indicating relatively thicker measurements in the nasal relative to the temporal retina (Figure 3 and Figure S1). These spatial patterns closely resembled the distribution of retinal ganglion cells across the retina^75^ and GCL thickness measurements across the macula from OCT-based studies.^13,14,32^ Of note, there were three to four statistically separable clusters comprising GCIPL measurements outside the macular regions investigated in previous cluster analysis studies,^13,14,32^ indicating measurable differences in GCIPL thickness within different regions of the mid-peripheral retina.

### Regression models characterising age-related change in the GCIPL

Regression models were applied to characterise changes in the GCIPL across the widefield measurement area as a function of age. Given the overall similarities between cluster patterns derived from hierarchical and two-step cluster algorithms, results of regression modelling detailed below are for data pooled using the hierarchical cluster pattern. Sliding window analyses revealed significantly better fits using quadratic regression models relative to linear models across all but the peripheral-most clusters (*p* < 0.001–0.007, Table 3). However, closer investigation of adjusted *R*^2^ values revealed only small improvements in *R*^2^ up to 0.003 using quadratic models. Given the little meaningful improvement when applying the more complex quadratic regression models, linear regression models were subsequently applied to data pooled using the original age brackets and by cluster.

**TABLE 3:** Comparisons between quadratic and linear regression models applied to sliding window analyses, for data pooled by clusters derived from the hierarchical cluster pattern.

	Hierarchical			
	Adjusted *R*^2^		*F* change	*p* Value
	Quadratic	Linear		
Cluster 1	0.008	0.008	0.6659	0.42
Cluster 2	0.026	0.026	0.293	0.59
Cluster 3	0.084	0.083	7.4162	0.007
Cluster 4	0.146	0.144	13.261	0.0003
Cluster 5	0.206	0.203	19.364	<0.0001
Cluster 6	0.257	0.254	24.284	<0.0001
Cluster 7	0.289	0.285	28.88	<0.0001
Cluster 8	0.263	0.260	21.957	<0.0001
Cluster 9	0.238	0.235	17.165	<0.0001

*Note*: Clusters are labelled as per Figure 3, where increasing cluster number denotes increasing mean GCIPL thickness.

Abbreviations: GCIPL, ganglion cell–inner plexiform layer; *R*^2^, coefficient of determination.

Linear regression modelling revealed that significantly different rates of change in GCIPL thickness were observed across all clusters, when expressed as both raw GCIPL thickness (likelihood ratio test statistic 493.14 and F 19.19, *p* < 0.0001 for both) and as log(GCIPL thickness) to describe the percentage change with increasing age (likelihood ratio test statistic −170.89 and *F* 5.87, *p* < 0.0001 for both). Post-hoc comparisons of linear regression coefficients between clusters suggested that GCIPL thickness slopes were shared between Clusters 1 and 2, Clusters 3 and 4 and Clusters 5–9, while for log(GCIPL thickness), slopes were shared between Clusters 1 and 2 and Clusters 3–9 (Table S4). Additional F tests with clusters pooled for these groups confirmed non-significant differences in slopes using these cluster groupings for GCIPL thickness (*F* = 1.240, 2.824 and 1.119 and *p* = 0.27, 0.09 and 0.35 for Clusters 1 and 2, Clusters 3 and 4 and Clusters 5–9, respectively, Table 4 and Figure 3) and log(GCIPL thickness) (*F* = 2.476 and 1.173, and *p* = 0.12 and 0.32 for Clusters 1 and 2 and Clusters 3–9, respectively, Table 4). Overall, reductions in GCIPL thickness as a function of age were observed across the entire measurement area, although they were slower at the peripheral two clusters using both GCIPL thickness and log(GCIPL thickness) data, representative of slower absolute and percentage change in GCIPL thickness at these peripheral regions. Meanwhile, while significantly slower rates of change in GCIPL thickness were observed in Clusters 3 and 4 relative to the more central Clusters 5–9, differences were no longer appreciable when expressed as percentage change. For completeness, inter-cluster regression coefficient comparisons were also performed for data pooled using the two-step cluster pattern. While variable rates of change in GCIPL thickness with age could be appreciated between clusters, these differences disappeared when expressed as percentage change, which is largely similar to the results using the hierarchical cluster pattern (Tables S5 and S6).

**TABLE 4:** Coefficients and goodness of fit parameters for linear regression models applied to ganglion cell–inner plexiform layer (GCIPL) thickness measurements and log(GCIPL thickness) pooled by age bracket and clusters derived from the hierarchical cluster pattern.

	GCIPL thickness			Log(GCIPL thickness)		
	Equation	*R* ^2^	RMSE	Equation	*R* ^2^	RMSE
Cluster 1	−0.04*x + 32.89	0.007	5.46	−0.0005*x + 1.51	0.035	0.036
Cluster 2	−0.04*x + 38.53	0.024	5.46	−0.0005*x + 1.59	0.080	0.036
Cluster 3	−0.12*x + 52.06	0.079	5.47	−0.0010*x + 1.71	0.152	0.036
Cluster 4	−0.12*x + 62.57	0.140	5.48	−0.0010*x + 1.80	0.187	0.036
Cluster 5	−0.19*x + 75.23	0.198	5.50	−0.0010*x + 1.87	0.209	0.036
Cluster 6	−0.19*x + 83.76	0.255	5.49	−0.0010*x + 1.92	0.214	0.036
Cluster 7	−0.19*x + 91.97	0.285	5.50	−0.0010*x + 1.97	0.210	0.036
Cluster 8	−0.19*x + 99.68	0.266	5.48	−0.0010*x + 2.00	0.169	0.036
Cluster 9	−0.19*x + 106.6	0.244	5.48	−0.0010*x + 2.04	0.133	0.036

*Note*: In each regression equation, x denotes participant age. Clusters were labelled as per Figure 3, where increasing cluster number denoted increasing mean GCIPL thickness.

Abbreviations: *R*^2^, coefficient of determination; RMSE, root mean square error.

## DISCUSSION

This study described the automated processing of GCIPL thickness measurements using deep learning methods from a widefield OCT protocol capturing data over the central 55° × 45° of the human retina; a larger area than any single volume scan protocol presently available on commercial instrumentation. In the studied healthy cohort, changes in GCIPL thickness as a function of age, axial length and sex were observed. Of note were the location-specific variations as a function of age, with a greater rate of decline observed in more central regions. By investigating factors contributing to normal variations in GCIPL thickness measurements from widefield OCT in detail, detection of quantitative change outside expected normative limits becomes possible, potentially allowing for precise, objective detection of inner retinal pathologies such as glaucoma. In conjunction with the ease of scan acquisition,^11^ the described widefield OCT protocol has promising clinical utility for both qualitative and quantitative assessments of the inner retina at non-central locations.

### Changes in widefield GCIPL thickness with demographic factors

The spatial patterns of age-related change in GCIPL thickness observable upon application of cluster analysis methods closely resembled distributions reported in histological studies of ganglion cells,^75^ in line with others reporting comparability between histological and OCT data.^22,52,76^ Moreover, the rates of GCIPL decline in macular OCT studies between −0.10 and −0.20 μm/year appear largely consistent with the averaged slope parameters across clusters of −0.14 μm/year in the hierarchical cluster pattern and −0.12 μm/year in the two-step cluster pattern.^15,17,20,77^ Interestingly, while the observation of faster rates of GCIPL decline centrally than peripherally with location-specific analyses was similar to that of previous studies applying cluster analysis to the macular GCL,^13,14,32^ differences in rates of decline within the macular region were minimised when expressed as percentage change per year, which was also observed in Tong et al.^13^ Moreover, these studies also reported significant improvements in non-linear models in characterising rates of change in the GCL. While this study also found significant improvements on the application of quadratic regression models, the relatively small improvement in model fit across clusters and cluster patterns did not appear to justify the application of this more complex model, and the potential for overfitting may increase the risk of reduced generalisability of this model to other datasets.^78^ Additionally, part of this difference may be due to the use of the GCIPL in this study, which was chosen due to difficulties in delineating the GCL-IPL boundary. While Trinh et al.^32^ described quadratic regression models of age-related decline in IPL thickness, the slower rate of decline compared to co-localised GCL may mask some trends in regression data.

The additional findings of significant decrease in global GCIPL thickness with increasing axial length and female sex were similar to those reported in macular OCT studies investigating demographic variables influencing the GCIPL,^17,20,21^ suggesting that these trends persist in widefield investigations of the GCIPL. In particular, the association of increasing axial length with decreasing GCIPL thickness in peripheral macular locations appears consistent with the present findings,^21,50^ given the greater retinal area imaged using widefield OCT. While the magnitude of this effect was reduced with adjustment of transverse magnification,^50^ suggesting axial length-induced magnification effects contributed in part to these observations, the persistence of these trends with correction for axial length plus reported correlations between GCL volume adjusted for axial length and optic chiasm volume^79^ may indicate axial length-dependent variations independent of magnification. Perhaps it is not surprising therefore that with the inclusion of more peripheral retinal locations in the widefield OCT scan, the significant association persists.

### Comparison with other widefield OCT protocols

**FIGURE 4:**
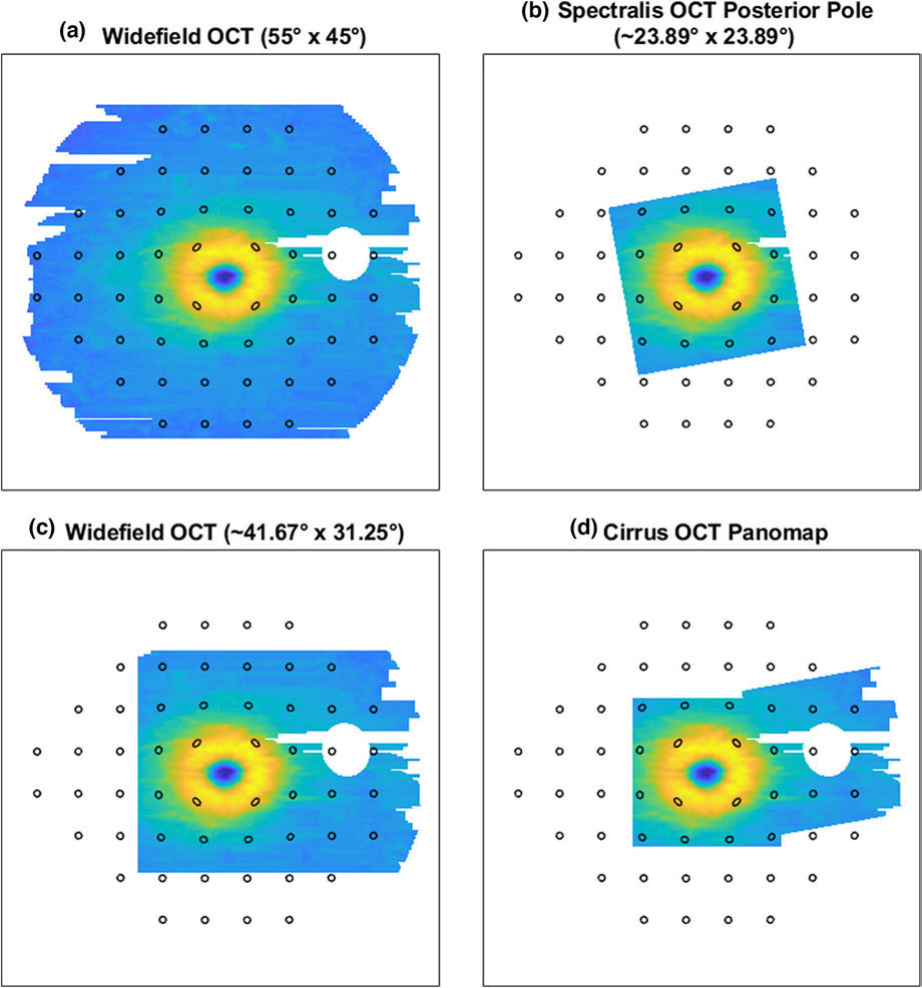
Comparison of projected 24-2 visual field locations (black ellipses) and various ocular coherence tomography (OCT) protocols. The slight distortion of the central 16 visual field test locations is in line with the lateral displacement of retinal ganglion cells.^86,87^ (a) The widefield OCT protocol described in this study, which captured the majority of visual field test locations. (b) The posterior pole volume scan area per Spectralis OCT, as depicted in Figure 3. (c) The widefield OCT protocol described in several studies,^5,7–9,53,88^ primarily using the Topcon DRI OCT Triton. (d) The Cirrus OCT Panomap function, which montages individually acquired macular and optic disc scans. Dimensions for the Cirrus OCT Panomap are not provided as this depends on overlap between individual macular and optic disc scans. Note that for c and d, displacement of the scan to capture both the macula and optic disc resulted in fewer OCT data points corresponding to the superior, temporal and inferior visual field.

The widefield OCT acquisition protocol utilised in this study and the resultant derived normative models vary from those described by others in several ways. First, while widefield RNFL OCT scans have been evaluated in the detection of glaucomatous arcuate defects and the development of widefield normative databases,^7,8,53,80^ given the need to consider variations in RNFL trajectory and peripapillary vasculature in RNFL analyses,^30,53,81^ inter-individual comparisons of GCIPL thickness are generally less complex. Moreover, the 55° × 45° scanning protocol captured a greater area of the temporal, superior and inferior retina relative to other widefield protocols. Not only could the larger field of view facilitate qualitative detection of extra-macular retinal pathology,^82^ it was also more comparable to the retinal area sampled using visual field paradigms commonly used in clinical practice (Figure 4). Due to instrument-related limitations, clinical comparisons of structure and function are often based on extrapolated RNFL projections, which was associated with some degree of error due to associated inter-individual variation.^30,83,84^ By capturing retinal information over the area sampled during the assessment of visual fields, widefield OCT protocols may facilitate clinical interpretations of structure–function concordance by enabling more direct comparisons between OCT and visual field data. Indeed, studies using montaged OCT scans over the 24-2 visual field area revealed moderate correlations between VF and OCT data,^29,85^ indicating the potential of widefield OCT given the equivalent measurement area but faster and easier scan acquisition.^11^

### Clinical implications of widefield OCT in detecting inner retinal pathology

With the identification of factors influencing normal variations in GCIPL thickness and incorporation into a normative database, identification of abnormal thickness measurements can be reliable, automatic and easy to implement in the clinical setting, akin to macular OCT's utility in detecting GCIPL loss in glaucoma.^2,40^ Moreover, given known increases in inherent variability in visual field results with more advanced disease,^10,89–91^ widefield OCT may become incredibly powerful to detect regions of glaucomatous damage outside the macular region objectively, particularly when questionable reliability on functional testing is observed.

However, some potential limitations of the widefield OCT should be considered prior to clinical implementation. First, while this study demonstrated that the automated segmentation algorithm was highly repeatable, manual confirmation by clinicians of correct boundary delineation would be prudent, as performed in this study. Widefield OCT may also be affected by measurement floor effects at more peripheral locations.^40^ The three to four distinct extra-macular clusters (Figure 3) indicated that measurable differences in GCIPL thickness occurred with increasing eccentricity, and therefore there may be reasonable reductions in GCIPL thickness at retinal locations affected by glaucoma. Moreover, extrapolating from previous work suggesting that glaucomatous visual field loss can occur with GCIPL measurements at the 15th percentile distribution limits of normative data in the peripheral macula,^40^ this corresponded to a reduction in GCIPL thickness of 2.11–2.60 μm, or 5.80%–7.08% at the extra-macular clusters (Table S7). Future studies applying the described widefield OCT protocol on glaucomatous eyes would be valuable to investigate further the clinical potential of this relatively novel imaging protocol.

### Limitations

There were several limitations to this study. First, the paucity of participants identifying as ethnicities other than White or East Asian precluded more detailed analyses of the potential impacts of ethnicity on widefield GCIPL thicknesses. Given previous reports of potential ethnicity-based variations,^15,17^ investigations including a more ethnically diverse cohort are warranted to confirm how ethnicity may impact GCIPL thickness. Furthermore, the resolution of the widefield OCT scanning protocol was not sufficient to visualise the temporal raphe, and therefore this study did not account for variations in GCIPL thickness related to raphe position. Additionally, intra- and inter-visit repeatability of the widefield OCT protocol was not investigated in this study, which would be important to clarify the potential clinical utility of this OCT protocol. Finally, the cross-sectional study design was chosen to characterise GCIPL thickness variations over a large age range, with the caveat that these may not be entirely representative of intra-individual ageing changes; longitudinal studies are warranted to confirm the rate of change in GCIPL thickness.

## CONCLUSION

This study described the generation of GCIPL thickness measurements from a widefield OCT protocol more closely matching retinal locations sampled during conventional visual field assessment and characterised variations in resultant GCIPL thickness from a healthy cohort. Decreases in global GCIPL thickness were observed with increasing axial length and female sex, while cluster analysis methods revealed a more rapid decline in GCIPL thickness at central relative to peripheral locations as a function of increasing age. Derivation of these factors may allow for quantitative detection of abnormal GCIPL measurements using widefield OCT, and this protocol has potential to facilitate detection of inner retinal pathologies outside areas captured using commercial OCT protocols, as well as enable more direct comparisons of structural and functional data.

## Supplementary Information

Supplementary file (DOCX 525 KB)
