# Supplementary material for: Characterisation of the normal human ganglion cell–inner plexiform layer using widefield optical coherence tomography
Source: Ophthalmic Physiol Opt. 2023 Nov 22;44(2):457–71. doi: 10.1111/opo.13255 (PMC12872676; doi:10.1111/opo.13255)
Supplement: Supplementary file 1 — Supplementary file (DOCX 525 KB) [file 44402_2024_4402020_MOESM1_ESM.docx]

**Supplementary materials for: Characterization of the normal human ganglion cell-inner plexiform layer using widefield optical coherence tomography**

Janelle Tong^1,2^, David Alonso-Caneiro^3,4^, Jason Kugelman^4^, Jack Phu^1,2,5,6,7^, Sieu K. Khuu^2^, Michael Kalloniatis^2,7^

1. Centre for Eye Health, University of New South Wales (UNSW), Sydney, New South Wales, Australia

2. School of Optometry and Vision Science, UNSW, Sydney, New South Wales, Australia

3. School of Science, Technology and Engineering, University of Sunshine Coast, Sunshine Coast, Queensland, Australia

4. Queensland University of Technology, Contact Lens and Visual Optics Laboratory, Centre for Vision and Eye Research, School of Optometry and Vision Science, Kelvin Grove, Queensland, Australia

5. Faculty of Medicine, University of Sydney, Sydney, New South Wales, Australia

6. Concord Clinical School, Concord Repatriation General Hospital, Sydney, New South Wales, Australia

7. School of Medicine (Optometry), Deakin University, Waurn Ponds, Victoria, Australia

Supplementary Methods: 3

Supplementary Figures: 1

Supplementary Tables: 7

Corresponding author:

Prof Michael Kalloniatis

School of Medicine (Optometry), Deakin University

Waurn Ponds, Victoria, Australia 3216

Email: michael.kalloniatis@deakin.edu.au

**Supplementary Methods 1.** Technical details on the deep learning segmentation procedure

The deep learning approach utilized to segment the retinal nerve fiber layer-ganglion cell layer (RNFL-GCL) and inner plexiform layer-inner nuclear layer (IPL-INL) boundaries involving a fully semantic deep neural network (U-Net) followed by a graph search.^1, 2^ The U-Net was trained on a separate dataset consisting of 463 posterior pole volume scans from healthy eyes. Images from this dataset were divided into training, validation and testing sets by volume using a 60-20-20% split. The original OCT images (768x496 pixels) were divided into 6 non-overlapping slices of size 128x496 pixels to facilitate generalizability to different sized OCT scans, including those from widefield OCT (1536x496 pixels). The network was trained using the Adam optimizer (with default parameters) to minimise Dice loss between the target area mask and the area mask predicted by the network. The network was trained using a batch size of 32 slices for 100 epochs and model selection was performed using the epoch with the highest Dice coefficient on the validation set.

U-Net incorporates skip connections between the encoder and each corresponding layer of the decoder, and uses four max pooling layers, each with three convolution blocks (kernel sizes 1x1, 3x3, 3x3) consisting of one convolution (all stride 1), ReLU and batch normalization. A residual connection was added between the output of the first and third blocks, followed by a squeeze and excite module (scSE variant). Each up-sampling layer used nearest neighbor up-sampling followed by a 2x2 convolution block and two 3x3 convolution blocks (all stride 1).The input of each convolution layer was zero padded to match output and input size, and each convolution block in the first layer of the encoder, and corresponding last layer of the decoder, used 8 filters which doubled in each subsequent layer of the network. The output of the U-Net was the per-pixel classification into one of three classes, representing the regions separated by the RNFL-GCL and IPL-INL boundaries.

**Supplementary Methods 2.** Correlation analyses between axial length and refractive error

To determine whether axial length could be estimated from refractive error, correlation analyses were performed using SPSS Statistics, version 27.0 (IBM Corp, Armonk, NY, USA) on 329 participants for which axial length data were available and without a prior history of refractive surgery, including cataract surgery (70.06% of the total cohort). Pearson correlations were overall moderate (R = -0.647, P <0.001), and the slightly poorer Spearman’s rho in the context of data following a normative distribution suggested a linear model would best fit this data (rho = -0.622, P <0.001). As such, the subsequent linear regression model was used to estimate axial length from refractive error in the 125 participants without axial length information (26.54% of the total cohort), to enable further OCT data processing:

$$axial length= -0.412\times rx+23.757$$

Where rx denotes the spherical equivalent refractive error in diopters.

***Supplementary Methods Table 1.*** *Demographic characteristics on the sub-cohort with axial length data available.*

| **n** | **Age (y ± SD)** | **Sex**  **(M:F)** | **SE**  **(D ± SD)** | **AL (mm ± SD)** | **Eye (OD:OS)** | **Tilt (° ± SD)** | **IOP (mmHg ± SD)** | **Ethnicity (White:East Asian:Other)** |
| --- | --- | --- | --- | --- | --- | --- | --- | --- |
| 329 | 51.29 ± 17.54 | 152:193 | -0.42 ± 1.88 | 24.02 ± 1.21 | 170:159 | 7.18 ± 3.96 | 14.74 ± 3.20 | 171:100:58 |
|  |  |  |  |  |  |  |  |  |

*n, number of participants; y, years of age; SD, standard deviation; M, male; F, female; SE, spherical equivalent refractive error; D, diopters; AL, axial length; mm, millimeters; OD, right eye; OS, left eye; °, degrees; IOP, intraocular pressure*


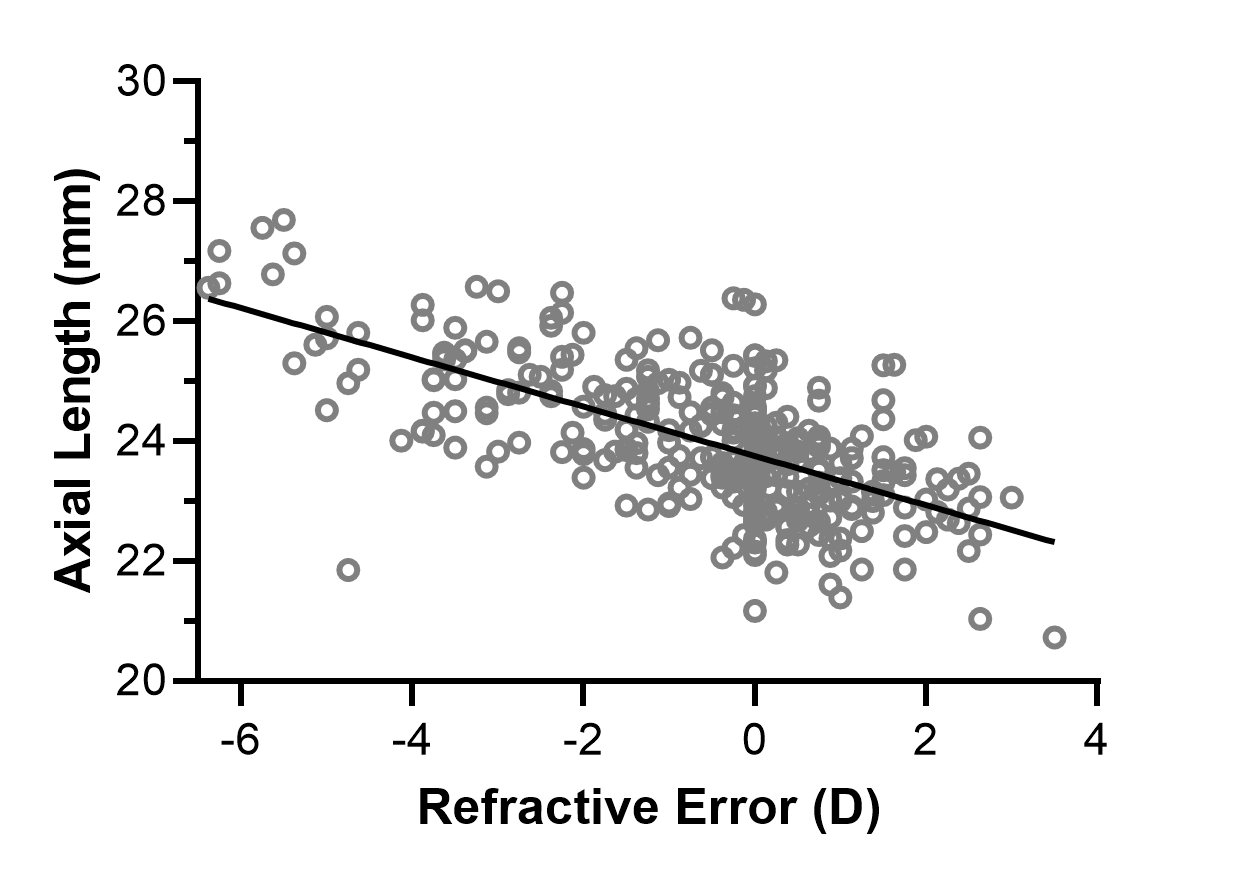


***Supplementary Methods Figure 1.*** *Linear regression model of refractive error in diopters (D) versus axial length in millimeters (mm).*

**Supplementary Methods 3.** Corrected GCIPL thickness measurements based on retinal tilt

Differences between uncorrected and corrected GCIPL thicknesses as a function of retinal tilt were derived from a randomly selected sub-cohort of 60 participants (**Supplementary Table 1**), due to the processing power required to calculate perpendicular retinal thicknesses across the entire widefield OCT scanning area. Methods applied were similar to those described in Tong et al.^3^, but automated using Matlab Version 2021b to capture the entire scan area. Axial GCIPL thickness and tilt of the IPL-INL boundary relative to the fovea center were calculated across the entire scan area. At each location, the corrected GCIPL thickness was calculated as the straight line distance between the intersection between the RNFL-GCL boundary and a straight line drawn perpendicular to the IPL-INL boundary. Differences between the corrected and uncorrected GCIPL thickness measurements were plotted as a function of retinal tilt, and Matlab Curve Fitting Toolbox™ (Mathworks, Natick, MA, USA) was used to derive the resultant quadratic regression model (adjusted R^2^ = 0.8761):

$$difference= -14.03\times tilt^{2}+1.611\times tilt-0.07437$$

Where thickness difference is in micrometers and retinal tilt is in radians. This equation was applied across the entire cohort to correct GCIPL thickness for retinal tilt for subsequent analyses.


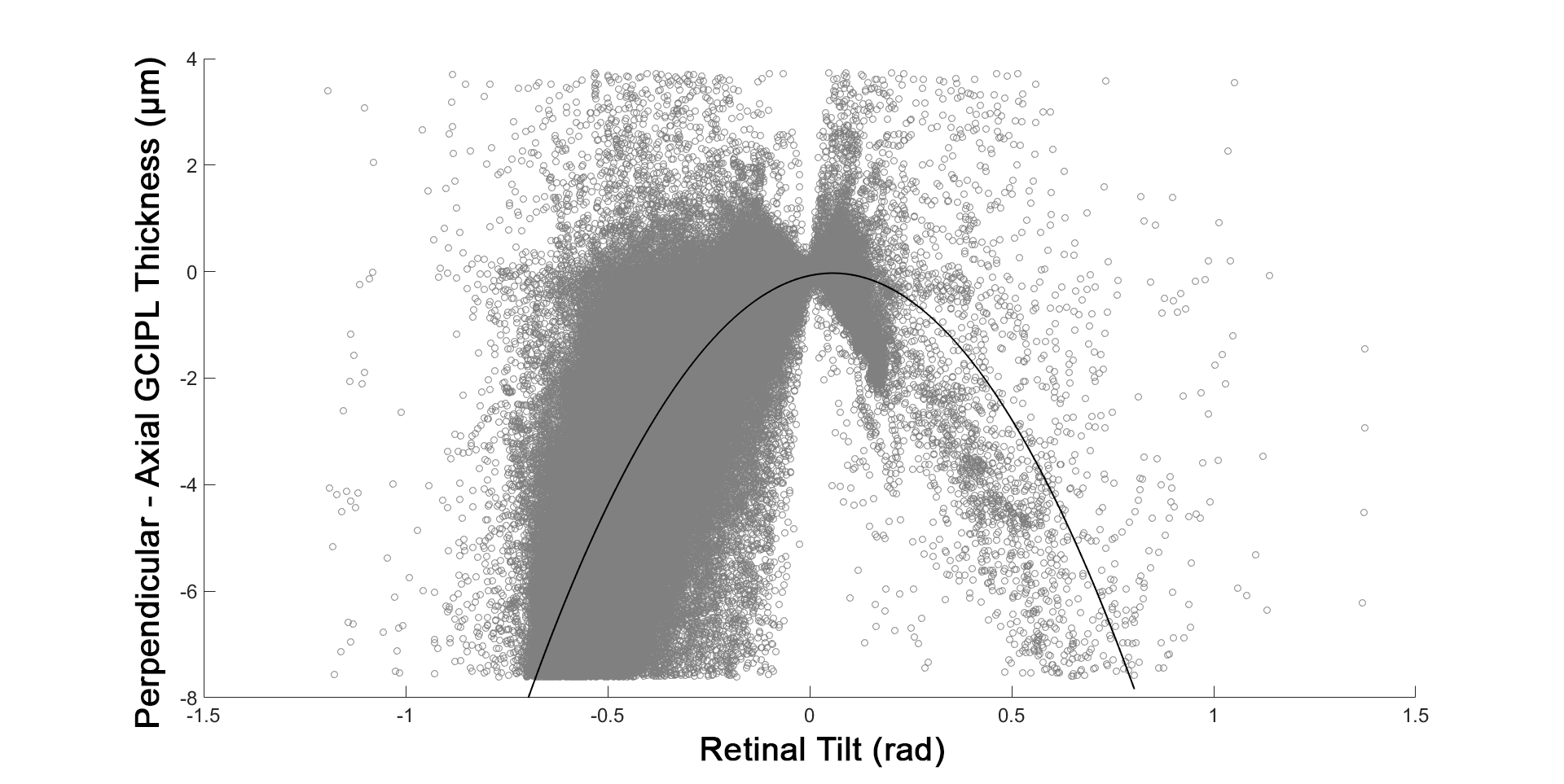


***Supplementary Methods Figure 2.*** *Quadratic regression model of retinal tilt relative to the foveal center in radians (rad) versus the difference in perpendicular and axial GCIPL thickness in microns (μm).*


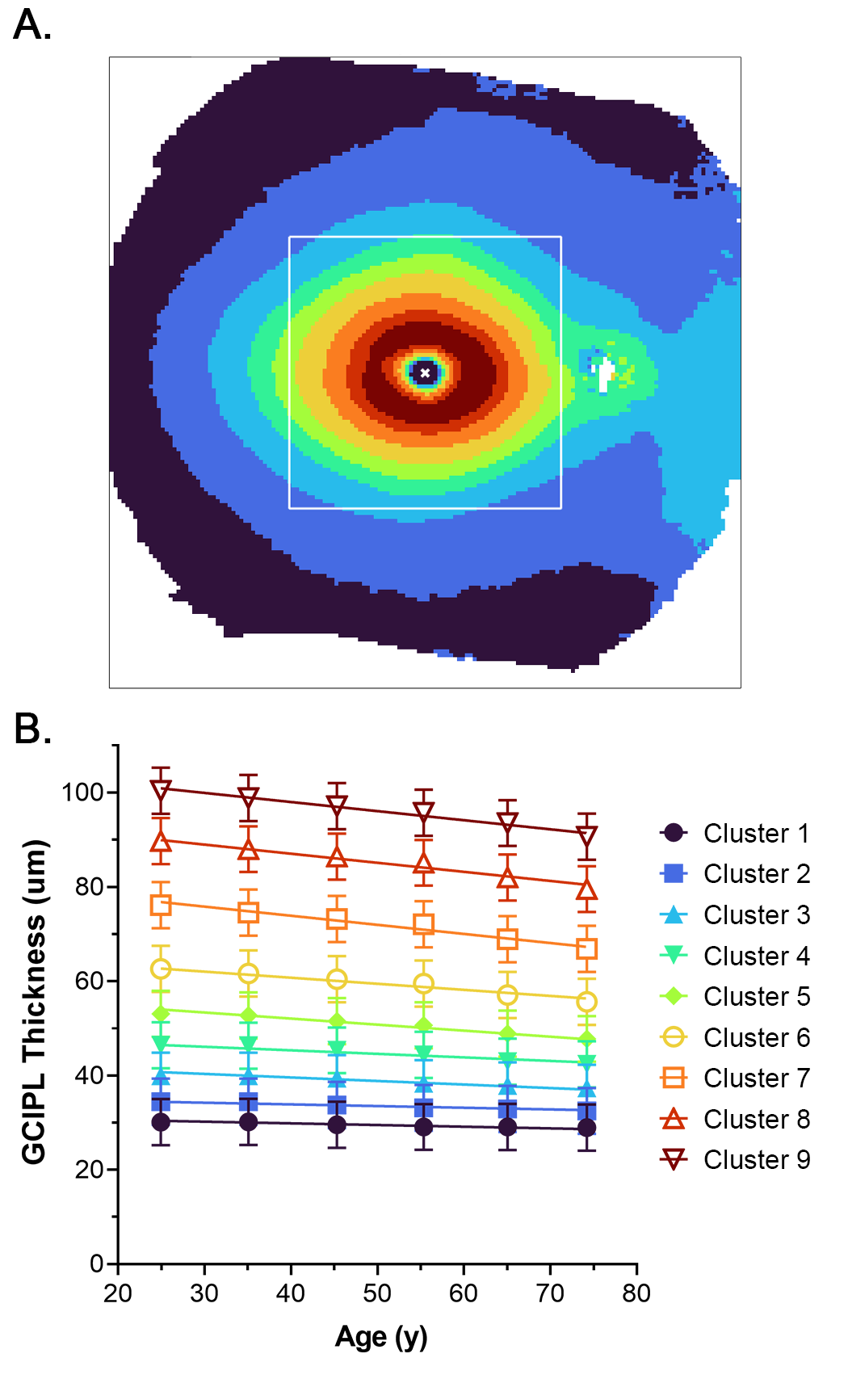


**Supplementary Figure 1.** A. Spatial patterns of age-related change in GCIPL thickness across the central 55° x 45° of the retina, generated using the two-step cluster algorithm. Different colors denote statistically separable clusters of increasing GCIPL thickness. The white box demarcates the posterior pole volume scan area using the Spectralis OCT to enable visualization of the additional retinal area captured using widefield OCT. B. Linear regression model describing GCIPL thickness pooled by cluster as a function of age. The differently colored regression lines match the colored locations in A. Equations and fit parameters can be found in Supplementary Table 6.

**Supplementary Table 1.** Demographic characteristics on the sub-cohort that was used to derive corrected GCIPL thickness measurements based on retinal tilt and comparisons of measured versus calculated axial length.

| **n** | **Age (y ± SD)** | **Sex**  **(M:F)** | **SE**  **(D ± SD)** | **AL (mm ± SD)** | **Eye (OD:OS)** | **Tilt (° ± SD)** | **IOP (mmHg ± SD)** | **Ethnicity (White:East Asian:Other)** |
| --- | --- | --- | --- | --- | --- | --- | --- | --- |
| 60 | 51.00 ± 17.87 | 31:29 | -0.47 ± 2.04 | 24.03 ± 1.44 | 35:25 | 6.40 ± 3.70 | 14.89 ± 3.15 | 30:17:13 |

n, number of participants; y, years of age; SD, standard deviation; M, male; F, female; SE, spherical equivalent refractive error; D, diopters; AL, axial length; mm, millimeters; OD, right eye; OS, left eye; °, degrees; IOP, intraocular pressure

**Supplementary Table 2.** Distribution of self-reported ethnicities within the study cohort.

| **Ethnicity** | **Number (%)** |
| --- | --- |
| White | 240 (51.06) |
| East Asian | 143 (30.43) |
| Aboriginal or Pacific Islander | 3 (0.64) |
| African | 1 (0.21) |
| Central or South American | 24 (5.10) |
| South Asian | 18 (3.83) |
| Middle Eastern | 21 (4.47) |
| Mixed | 20 (4.26) |

%, percentage

**Supplementary Table 3.** Multiple linear regression analyses including participants self-identifying as White or East Asian only, to investigate the potential effect of ethnicity in addition to the other variables per Table 2.

| **Variable** | **Parameter Estimate ± SE** | **P value** |
| --- | --- | --- |
| Age | -0.058 ± 0.009 | <0.001 |
| Axial length | -0.478 ± 0.133 | <0.001 |
| Sex | -0.502 ± 0.298 | 0.092 |
| Ethnicity | 0.043 ± 0.294 | 0.883 |

SE, standard error

**Supplementary Table 4.** P values from Tukey’s multiple comparisons test comparing slope coefficients between cluster-specific linear regression models using GCIPL thickness data and log(GCIPL thickness), pooled per the hierarchical cluster pattern (Figure 2). For GCIPL thickness, results suggested that Clusters 1 and 2 shared the same slope coefficient, as did Clusters 3 and 4 and Clusters 5 to 9. For log(GCIPL thickness), results suggested that Clusters 1 and 2 shared the same slope coefficient, as did Clusters 3 to 9. These were subsequently tested using additional F tests, detailed in the main manuscript.

| GCIPL Thickness | | 1 | | 2 | | 3 | | 4 | | 5 | | 6 | | 7 | | 8 | |
| --- | --- | --- | --- | --- | --- | --- | --- | --- | --- | --- | --- | --- | --- | --- | --- | --- | --- |
| 2 | | 0.973 | |  | |  | |  | |  | |  | |  | |  | |
| 3 | | 0.038 | | 0.486 | |  | |  | |  | |  | |  | |  | |
| 4 | | <0.0001 | | 0.005 | | 0.760 | |  | |  | |  | |  | |  | |
| 5 | | <0.0001 | | <0.0001 | | 0.041 | | 0.862 | |  | |  | |  | |  | |
| 6 | | <0.0001 | | <0.0001 | | 0.0004 | | 0.139 | | 0.948 | |  | |  | |  | |
| 7 | | <0.0001 | | <0.0001 | | <0.0001 | | 0.013 | | 0.527 | | 0.997 | |  | |  | |
| 8 | | <0.0001 | | <0.0001 | | 0.0001 | | 0.069 | | 0.854 | | >0.999 | | >0.999 | |  | |
| 9 | | <0.0001 | | <0.0001 | | 0.001 | | 0.249 | | 0.988 | | >0.999 | | 0.981 | | >0.999 | |
| Log(GCIPL thickness) | 1 | | 2 | | 3 | | 4 | | 5 | | 6 | | 7 | | 8 | |  |
| 2 | 0.821 | |  | |  | |  | |  | |  | |  | |  | |  |
| 3 | 0.014 | | 0.594 | |  | |  | |  | |  | |  | |  | |  |
| 4 | 0.001 | | 0.120 | | 0.995 | |  | |  | |  | |  | |  | |  |
| 5 | <0.0001 | | 0.023 | | 0.887 | | >0.999 | |  | |  | |  | |  | |  |
| 6 | <0.0001 | | 0.014 | | 0.8207 | | 0.999 | | >0.999 | |  | |  | |  | |  |
| 7 | <0.0001 | | 0.023 | | 0.887 | | >0.999 | | >0.999 | | >0.999 | |  | |  | |  |
| 8 | 0.003 | | 0.309 | | >0.999 | | >0.999 | | 0.986 | | 0.968 | | 0.986 | |  | |  |
| 9 | 0.055 | | 0.856 | | >0.999 | | 0.936 | | 0.644 | | 0.544 | | 0.644 | | 0.995 | |  |

**Supplementary Table 5.** P values from Tukey’s multiple comparisons test comparing slope coefficients between cluster-specific linear regression models using GCIPL thickness data and log(GCIPL thickness), pooled per the two-step cluster pattern (Supplementary Figure 1). For GCIPL thickness, results suggested that Clusters 1 and 2 shared the same slope coefficient, as did Clusters 3 and 4, Clusters 5 and 6 and Clusters 7 to 9. For log(GCIPL thickness), results suggested that Clusters 1 to 3 shared the same slope coefficient, as did Clusters 4 to 9. These were subsequently tested in additional F tests (Supplementary Table 6).

| GCIPL Thickness | | 1 | | 2 | | 3 | | 4 | | 5 | | 6 | | 7 | | 8 | |
| --- | --- | --- | --- | --- | --- | --- | --- | --- | --- | --- | --- | --- | --- | --- | --- | --- | --- |
| 2 | | 0.995 | |  | |  | |  | |  | |  | |  | |  | |
| 3 | | 0.792 | | 0.998 | |  | |  | |  | |  | |  | |  | |
| 4 | | 0.022 | | 0.236 | | 0.725 | |  | |  | |  | |  | |  | |
| 5 | | 0.0003 | | 0.009 | | 0.100 | | 0.971 | |  | |  | |  | |  | |
| 6 | | <0.0001 | | <0.0001 | | 0.0002 | | 0.127 | | 0.781 | |  | |  | |  | |
| 7 | | <0.0001 | | <0.0001 | | <0.0001 | | <0.0001 | | 0.004 | | 0.415 | |  | |  | |
| 8 | | <0.0001 | | <0.0001 | | <0.0001 | | <0.0001 | | <0.0001 | | 0.053 | | 0.993 | |  | |
| 9 | | <0.0001 | | <0.0001 | | <0.0001 | | <0.0001 | | 0.002 | | 0.313 | | >0.999 | | 0.998 | |
| Log(GCIPL thickness) | 1 | | 2 | | 3 | | 4 | | 5 | | 6 | | 7 | | 8 | |  |
| 2 | 0.964 | |  | |  | |  | |  | |  | |  | |  | |  |
| 3 | 0.692 | | >0.999 | |  | |  | |  | |  | |  | |  | |  |
| 4 | 0.013 | | 0.306 | | 0.723 | |  | |  | |  | |  | |  | |  |
| 5 | 0.002 | | 0.103 | | 0.391 | | >0.999 | |  | |  | |  | |  | |  |
| 6 | 0.0001 | | 0.015 | | 0.0986 | | 0.971 | | 0.999 | |  | |  | |  | |  |
| 7 | <0.0001 | | 0.002 | | 0.018 | | 0.754 | | 0.956 | | >0.999 | |  | |  | |  |
| 8 | 0.0001 | | 0.017 | | 0.110 | | 0.977 | | >0.999 | | >0.999 | | >0.999 | |  | |  |
| 9 | 0.020 | | 0.388 | | 0.803 | | >0.999 | | >0.999 | | 0.944 | | 0.669 | | 0.954 | |  |

**Supplementary Table 6.** Coefficients and goodness of fit parameters for linear regression models applied to GCIPL thickness measurements and log(GCIPL thickness) pooled by age bracket and clusters derived from the hierarchical cluster pattern. In each regression equation, x denotes participant age. Clusters are labelled as per Supplementary Figure 1, where increasing cluster number denotes increasing mean GCIPL thickness.

|  | **GCIPL Thickness** | | | **Log(GCIPL thickness)** | | |
| --- | --- | --- | --- | --- | --- | --- |
|  | **Equation** | **R^2^** | **RMSE** | **Equation** | **R^2^** | **RMSE** |
| Cluster 1 | -0.04*x+31.27 | 0.008 | 4.87 | -0.0005*x+1.50 | 0.031 | 0.036 |
| Cluster 2 | -0.04*x+35.29 | 0.021 | 4.86 | -0.0005*x+1.55 | 0.066 | 0.036 |
| Cluster 3 | -0.07*x+42.57 | 0.035 | 4.87 | -0.0005*x+1.62 | 0.084 | 0.036 |
| Cluster 4 | -0.07*x+48.32 | 0.086 | 4.88 | -0.001*x+1.70 | 0.148 | 0.036 |
| Cluster 5 | -0.13*x+57.22 | 0.127 | 4.88 | -0.001*x+1.75 | 0.169 | 0.036 |
| Cluster 6 | -0.13*x+65.88 | 0.193 | 4.89 | -0.001*x+1.82 | 0.195 | 0.036 |
| Cluster 7 | -0.19*x+81.59 | 0.286 | 4.90 | -0.001*x+1.91 | 0.215 | 0.036 |
| Cluster 8 | -0.19*x+94.75 | 0.323 | 4.90 | -0.001*x+1.98 | 0.193 | 0.036 |
| Cluster 9 | -0.19*x+105.7 | 0.294 | 4.89 | -0.001*x+2.03 | 0.143 | 0.036 |

R^2^, coefficient of determination; RMSE, root mean square error

**Supplementary Table 7.** GCIPL thickness measurements corresponding to increased likelihood of a co-localized glaucomatous visual field defect in extra-macular clusters (Figure 3), per a threshold cut-off of the 15% percentile limit of the normative data distribution.^4^ Normative data were age corrected to 51.31 years, the mean age of the total cohort, as well as for sex and axial length. Differences are expressed in both microns (µm) and as percentage reductions relative to mean GCIPL thickness.

|  | **Hierarchical** | | | **Two-step** | | |
| --- | --- | --- | --- | --- | --- | --- |
|  | **Mean GCIPL thickness (µm)** | **15^th^ percentile limit (µm)** | **Difference (µm, %)** | **Mean GCIPL thickness (µm)** | **15^th^ percentile limit (µm)** | **Difference (µm, %)** |
| Cluster 1 | 29.87 | 27.76 | 2.11 (7.08) | 28.63 | 27.07 | 1.56 (5.44) |
| Cluster 2 | 36.53 | 33.99 | 2.54 (6.95) | 33.27 | 31.81 | 1.46 (4.40) |
| Cluster 3 | 44.87 | 42.27 | 2.60 (5.80) | 38.70 | 36.81 | 1.89 (4.89) |
| Cluster 4 | **-** | **-** | **-** | 44.52 | 42.45 | 2.07 (4.65) |

**REFERENCES FOR SUPPLEMENTARY MATERIAL**

1. Kugelman J, Allman J, Read SA, et al. A comparison of deep learning U-Net architectures for posterior segment OCT retinal layer segmentation. *Sci Rep* 2022;12(1):14888.

2. Kugelman J, Alonso-Caneiro D, Read SA, et al. Automatic choroidal segmentation in OCT images using supervised deep learning methods. *Sci Rep* 2019;9(1):13298.

3. Tong J, Yoshioka N, Alonso-Caneiro D, Zangerl B. Ganglion cell-inner plexiform layer measurements derived from widefield compared to montaged 9-field optical coherence tomography. *Clin Exp Optom* 2021; doi:10.1080/08164622.2021.1993058:1-9.

4. Tong J, Alonso-Caneiro D, Kalloniatis M, Zangerl B. Prediction of visual field defects from macular optical coherence tomography in glaucoma using cluster analysis. *Ophthalmic Physiol Opt* 2022;42(5):948-64.
